# Effects of Acetone Fraction From *Buchenavia tomentosa* Aqueous Extract and Gallic Acid on *Candida albicans* Biofilms and Virulence Factors

**DOI:** 10.3389/fmicb.2018.00647

**Published:** 2018-04-05

**Authors:** Guilherme R. Teodoro, Aline V. L. Gontijo, Marcos J. Salvador, Márcia H. Tanaka, Fernanda L. Brighenti, Alberto C. B. Delbem, Ádina C. B. Delbem, Cristiane Y. Koga-Ito

**Affiliations:** ^1^Environmental Engineering Department and Oral Biopathology Graduate Program, Institute of Science and Technology of São José dos Campos, São Paulo State University, São Paulo, Brazil; ^2^Department of Plant Biology, PPGBTPB, Institute of Biology, University of Campinas, Campinas, Brazil; ^3^Araquara Faculty of Dentistry, São Paulo State University, São Paulo, Brazil; ^4^Araçatuba Faculty of Dentistry, São Paulo State University, São Paulo, Brazil

**Keywords:** phytotherapy, antifungal, *Candida albicans*, *Buchenavia tomentosa*, gallic acid, virulence factors

## Abstract

A promising anti-*Candida* activity of *Buchenavia tomentosa* extracts was recently described. In the present work, experiments were carried out to determine the fraction with higher antifungal activity from a *B. tomentosa* extract. Acetone fraction (AF) was obtained from the aqueous extract from dried leaves (5 min/100°C) and it was the most effective one. Gallic acid (GA) was identified by electrospray ionization mass spectrometry (ESI–MS) and also chosen to perform antifungal tests due to its promising activity on *Candida albicans*. Minimal inhibitory and fungicidal concentrations (MIC and MFC) were determined by broth microdilution technique. The effect on virulence factors of *C. albicans* was evaluated, and the cytotoxicity was determined. MIC_50_ and MIC_90_ values were both equal to 0.625 mg ml^-1^ for AF and 2.5 and 5 mg ml^-1^, respectively, for GA. AF and GA showed ability to inhibit *C. albicans* adherence and to disrupt 48 h-biofilm. AF and GA were effective in reducing the formation of hyphae of *C. albicans* SC5314. AF and GA decreased adherence of *C. albicans* to oral epithelial cells. AF and GA showed slight to moderate toxicity to Vero cells. This result suggests further studies for topic use of these compounds. AF, which contains a combination of several molecules, presented greater potential of antimicrobial activity than GA, with lower values of MIC and lower cytoxicity.

## Introduction

*Candida* belongs to a vast and heterogeneous genus, presenting more than 200 species (Williams et al., 2013). The genus *Candida* presents many species, however only 20 are usually related to human infections (Williams et al., 2013), which range from superficial to systemic diseases. Superficial infectious, although less harmful, are more frequent and can be recurrent for some patients. Systemic infections initiate when fungi invade the epithelial barrier and reach the blood circulation, causing candidemia (Gow et al., 2012). The attributable mortality rate (25–60%) of patients with candidemia varies from 25 to 60%, with significant higher rates among developing countries (Kaur and Chakrabarti, 2017). *Candida albicans* is usually the most frequently involved species in cases of candidemia (Arendrup, 2013; da Matta et al., 2017; Fu et al., 2017).

*Candida albicans* expresses some virulence factors, which are important to initiate and establish the infectious process. The ability to form biofilms is one of its major virulence factors (Uppuluri et al., 2009). Biofilms are defined as a community of cells attached to a biotic or abiotic surface and covered by a polymer matrix (Donlan and Costerton, 2002). Such matrix protects the fungal cells from antimicrobial drugs, which makes biofilms very difficult to treat. There are two usual ways to treat biofilms. The first one is preventing the biofilm formation, avoiding its adherence or maturation (Girardot and Imbert, 2016). The second consists in the eradication of biofilm (Girardot and Imbert, 2016). Ideally, a good candidate to treat *Candida* biofilms should present both mechanisms.

Another virulence factor of *C. albicans* is the ability of changing from yeast cells to hyphae (Gow et al., 2012). This is a crucial step for the invasion of the epithelial barrier. The hyphal morphologies are believed to induce more tissue damage than yeast cells by producing and secreting lytic enzymes, such as secreted aspartyl proteinases (SAPs) (Naglik et al., 2003; Dalle et al., 2010).

Traditional antifungal drugs, such as azoles, amphotericin B, and echinocandin are generally used for the treatment of candidiasis (Pappas et al., 2004). Nonetheless, due to the limited number of antifungal agents and the misuse of the few available drugs, there are many cases of resistance among *Candida* species (Pappas et al., 2004). Natural products for the treatment of infections are promising, since they present lower toxicity with significant antimicrobial activity (Abreu et al., 2013).

*Buchenavia tomentosa* is a natural plant from the central region of Brazil which showed promising antifungal activity (Teodoro et al., 2015a; Brighenti et al., 2017). Although it is frequently used in the traditional medicine, there are only few studies in the scientific literature about the pharmaceutical applications of this plant.

In this context, the aims of this work were (i) to obtain different fractions of *B. tomentosa* leaves extracts and investigate their antifungal activity, (ii) to perform the chemical characterization of the most active fraction, (iii) to determine minimal inhibitory and fungicidal concentrations of this fraction, (iv) select one isolate compound with promising anti-candidal activity and determine inhibitory and fungicidal concentrations, and (v) to evaluate the effect of these antifungal agents on *C. albicans* virulence factors, determining their effect on biofilm formation and eradication, on the production of proteinase and phospholipase, on morphogenesis (hypha formation), and finally the *in vitro* activity on *C. albicans* adherence to oral epithelial cells.

## Materials and Methods

### Obtaining the Different Fractions From Aqueous Extracts of *Buchenavia tomentosa* Leaves

The powdered, air-dried leaves of *B. tomentosa* (Teodoro et al., 2015a) (20 g) were extracted by maceration at 100°C for 5 min with 400 mL water (Salvador et al., 2006). The aqueous extract was concentrated under vacuum in a rotary evaporator and was suspended in methanol (1:5 v/v). Then, the resulted suspension was partionated with hexane (1:1 v/v) and the corresponding hexanic fraction was removed before the addition of dichloromethane. Subsequently, the dichloromethane fraction was removed and the acetone solvent (1:1 v/v) was added. Finally, the acetone fraction (AF) was obtained. The fractions were dried and resuspended in aqueous solution. A flow chart with the process of obtaining of the different fractions is available in (**Supplementary Figure S1**).

### Screening of the Antifungal Activity of the Fractions

The methodology used for *in vitro* screening of the antifungal activity of the fractions was the double-layer agar technique. Reference strains of *C. albicans* (ATCC 18804 and SC 5314) were used. All four fractions obtained from the aqueous extract of *B. tomentosa* leaves were studied. The preparation of the substances and microorganisms was carried out as previously described (Teodoro et al., 2015a), with final concentration of the substances equal to 10 mg mL^-1^. Standardized fungal suspensions containing 10^6^ cells ml^-1^ were prepared and inoculated in RPMI 1640 agar. All the substances, except the AF, had 5% DMSO added.

### Chemical Characterization of Acetone Fraction (AF) From *B. tomentosa* Aqueous Extract

The characterization of the AF constituents was obtained by electrospray ionization mass spectrometry (ESI–MS) (Schinor et al., 2006). The samples were diluted in a solution containing 50% (v/v) chromatographic grade methanol, 50% (v/v) of a deionized water solution and 0.5% ammonium hydroxide (Merck, Darmstadt, Germany). The ESI–MS analyses were performed by direct injection in the Micromass^®^ quadrupole mass spectrometer (Waters Corporation, Milford, MA, United States), by electro-spray in negative and positive mode as previously described (Teodoro et al., 2015a). The constituents were identified by comparison of ESI–MS/MS spectra results with the authentic standard sample spectrum from the Laboratory of Pharmacognosy, Phytopharmaceutical Technology and Bioassays of IB-Unicamp, as well as with data from the literature.

### Determination of Minimum Inhibitory Concentration and Minimum Fungicidal Concentration of the Acetone Fraction (AF) and Gallic Acid (GA)

The reference strains *C. albicans* SC 5314 and ATCC 18804 were used, together with 27 previously obtained *C. albicans* clinical isolates from lesions of denture-associated erythematous candidiasis. The use of such strains was approved by the Local Human Research Ethics Committee (process # 367.923). The broth microdilution technique was performed as previously described (Teodoro et al., 2015a). Fungal suspensions containing 10^6^ cells ml^-1^ were prepared with the aid of a spectrophotometer. The initial concentration of AF and GA was transferred to 96-well plates and then was twofold serial diluted in 1640 RPMI broth (without sodium bicarbonate, with L-alanine, supplemented with 2% glucose and buffered in pH 6.5 with MOPS) to obtain a range of concentrations from 5 to 0.001 mg mL^-1^. After 24 h of incubation at 37°C, under aerobiosis, the results were determined by visual reading compared to the negative control. Amphotericin B (2 μg mL^-1^) was used as the positive control. Three independent experiments were performed in triplicate.

### Evaluation Against Virulence Factors of *Candida albicans*

#### Effect of Subinhibitory Concentration on *C. albicans* Biofilm Formation

The effect of AF and GA on biofilm formation was evaluated according to Capoci et al. (2015). *C. albicans* ATCC 18804, *C. albicans* SC 5314 and 3 clinical isolates were grown in Sabouraud dextrose agar and incubated at 37°C for 24 h. Standardized fungal suspensions containing 10^7^ cells ml^-1^ were obtained spectrophotometrically. In order to evaluate the effect of AF and GA on biofilm formation, 20 μl of fungal suspension was added to 180 μl of RPMI (final concentration of 10^6^ cells ml^-1^) with 2% glucose supplemented with AF and GA at subinhibitory concentrations (AF = 1.25 mg ml^-1^ and GA = 2.5 mg ml^-1^). The plates were incubated at 37°C under agitation (80 rpm) for 24 and 48 h. After this period, the biofilms were disrupted by vigorous vortexing followed by sonication. The suspensions were plated on Sabouraud dextrose agar. The plates were incubated at 37°C for 24 h and the number of colony forming units (CFUs) was obtained.

#### Effect on Preformed Biofilm

The methodology to evaluate the effect of AF and GA on pre-formed biofilm was based on Cheng et al. (2011), with some modifications. For this step, strains of *C. albicans* ATCC 18804, *C. albicans* SC 5314 and 3 clinical isolates were used. The 24 and 48 h biofilms were formed in polystyrene test specimens (50 mm^2^), previously sterilized by UV radiation for 30 min. The inoculums containing 10^7^ cells/ml were prepared with the aid of a spectrophotometer, as previously described (Teodoro et al., 2015a). 20 μl of the inoculums were then added to 180 μl of RPMI with 2% glucose in 96-well plates.

All plates were incubated for 120 min at 37°C at 80 rpm shaking for the pre-adhesion phase. The specimens were subsequently washed with 0.9% NaCl and transferred to new 96-well plates with 200 μl of RPMI with 2% glucose. After 24 or 48 h, the formed biofilms were exposed to 2 and 4 times MIC of tested substances for 5 min at room temperature. After this contact, the specimens were again washed and transferred to tubes containing 1 mL of 0.9% NaCl solution. The tubes were shaken for 60 s in vortex and also sonicated (2 pulses of 15 s with a 20 s interval, amplitude 40% and power of 15 W on ice) for biofilm disruption. The suspensions were plated on Sabouraud dextrose agar and incubated for 48 h at 37°C to obtain the values of CFUs/specimen.

#### Effect on the Production of Proteinase and Phospholipase

The formation of proteinase and phopholipase enzymes was evaluated in all the *C. albicans* strains described above (2 reference and 27 clinical isolates) after exposure to the tested substances. For this purpose, suspensions of 10^6^ cells ml^-1^ were prepared and exposed in aqueous solution of AF and GA at 1/2 MIC. The samples were incubated for 1 h at 37°C in 80 rpm rotation. As a positive control, PBS was used. The verification of proteinase and phospholipase production was made pipetting an aliquot of 2 μL of the treated strains into appropriate culture media.

The test medium for the evaluation of phospholipase production consisted of malt extract agar containing 1 M sodium chloride, 0.005 M calcium chloride and 2% egg yolk (bacto egg yolk enrichment 50%, 4 ml in 100 ml of agar) (Polak, 1992). For proteinase production assay, the test medium consisted of agar plates containing bovine serum albumin. 60 ml of a solution containing 0.04 g MgSO_4_. 7H_2_O, 0.5 g K_2_HPO_4_, 1 g NaCl, 0.2 g dried yeast extract, 4 g glucose, and 0.5 g BSA (fraction V) was prepared and the pH adjusted to 3.5 with 1 N HCl. This solution was sterilized by filtration and mixed with 140 ml of melted agar. After 4 days of incubation at 37°C, the enzymatic activity (Pz) was measured in terms of the ratio of the diameter of colony plus zone of halo, according to previous studies (Aoki et al., 1990; Polak, 1992). The Pz values were transformed into scores: (1) when the enzymatic activity was negative (Pz = 1), (2) when the activity was positive (Pz between 0.99 and 0.7) and (3) when the activity was strongly positive (≤0.69). The experiments were carried out in triplicate in three independent experiments.

#### Effect on Morphogenesis (Hyphae Formation)

The assays were performed according to Chevalier et al. (2012) with some modifications. Suspensions with 10^5^ cells/ml of *C. albicans* reference strains (ATCC 18804 and SC 5314) were prepared in RPMI containing AF and GA at 1/2 MIC. The negative control was RPMI without the substances. An aliquot of 1 ml of the preparations was transferred to 24-well plates and incubated at 37°C. Hyphae counts were performed using an optical microscope (200X) after 4 and 24 h of incubation at 37°C, including both true hyphae and pseudohyphae. Tests were performed in triplicate in three independent experiments.

### *In Vitro* Effect on *C. albicans* Adherence to Oral Epithelial Cells

The adherence of *C. albicans* to oral epithelial cells was performed according to Wellmer and Bernhardt (1997). The reference strains (ATCC 18804 and SC 5314) were plated on Sabouraud Dextrose agar and incubated at 37°C for 24 h. After the incubation, the cells were centrifuged (5000 × *g* for 3 min) and washed three times in 5 ml PBS. A 10^6^ cells ml^-1^ suspension was obtained as previously described. *C. albicans* cells were exposed to 1/2 MIC of AF and GA for 1 min. Following this, the yeasts were again centrifuged and washed with PBS. Epithelial cells were obtained from healthy volunteers, after signing the informed consent form (Local Ethical Committee protocol # 367.923), by scraping the jugal mucosa using sterile wooden spatulas. The obtained epithelial cells were centrifuged (10000 × *g* for 30 s) and washed three times with PBS. A suspension containing 10^5^ cells ml*^-^*^1^ was obtained by counting in Neubauer’s chamber. The aliquot of 100 μl of *C. albicans* blastoconidia and 100 μl of epithelial cell suspensions was mixed and incubated at 37°C for 1 h. Negative control (without treatment) was included. The mixture was filtered using membranes of 12 μm to eliminate the non-adhered *C. albicans* cells. Filters were washed with 0.5 ml of PBS and the contents of this wash were stained with crystal violet. The number of yeasts adhered in 25 epithelial cells was quantified.

### Cytotoxicity Assay

6 × 10^4^ Vero cells (fibroblast-like from kidney of green monkey) were seeded on 96-well plate and cultured under a temperature of 37°C and an atmosphere of 95% air and 5% CO_2_. Tests were carried out according to the International Organization of Standardization [ISO] (2009) 10993-5. The cells were grown in a cell culture medium containing Dulbecco’s modified Eagle’s medium (DMEM) supplemented with 5% of inactivated fetal bovine serum, 100 IU mL^-1^ of penicillin and 100 μg mL^-1^ of streptomycin and then incubated for 24 h to attach to the plate. AF and GA, at 0.625 and 1.25 mg ml^-1^, respectively, were incubated for 24 h. A volume of 100 μL of phosphate buffer solution (PBS) containing 0.5 mg ml^-1^ 3-(4,5-Dimethylthiazol-2-yl)-2,5-diphenyl tetrazolium bromide (MTT) was added and incubated for another 4 h. MTT solution was replaced by DMSO in order to dissolve the formazan crystals. The values of MTT-assay were measured using a microplate reader at 570 nm (Biotek Synergy^TM^ HT, Winooski, VT, United States). Non-treated cells were used as negative control. The percentage of cell viability was obtained considering the negative control as 100%. All the experiments were performed at least two times in triplicate.

### Statistical Analyses

The statistical analysis was performed with GraphPad Software, Inc. (San Diego, CA, United States) and a minimal level of significance of 5% was considered. The non-parametric Kruskal–Wallis or Friedman tests and Dunn’s multiple comparison *post hoc* test were applied since the distribution of data was not normal according to the D’Agostino & Pearson omnibus normality test.

## Results

### The Acetone Fraction Was the Most Promising Product

Four types of fractions from *B. tomentosa* aqueous extract were obtained: acetone, hydroalcoholic, hexane, and dichloromethane. The inhibition halos for fractions varied between 9 and 11 mm. The yield in relation to dried leaves was 13.2% for acetone, 0.7% for hydroalcoholic, 0.1% for hexane, and 0.04% for dichloromethane. The results showed that the most promising fraction was acetone, which presented the best yield. The AF was thus selected for further studies.

### Chemical Profile of the Acetone Fraction (AF)

**FIGURE 1:**
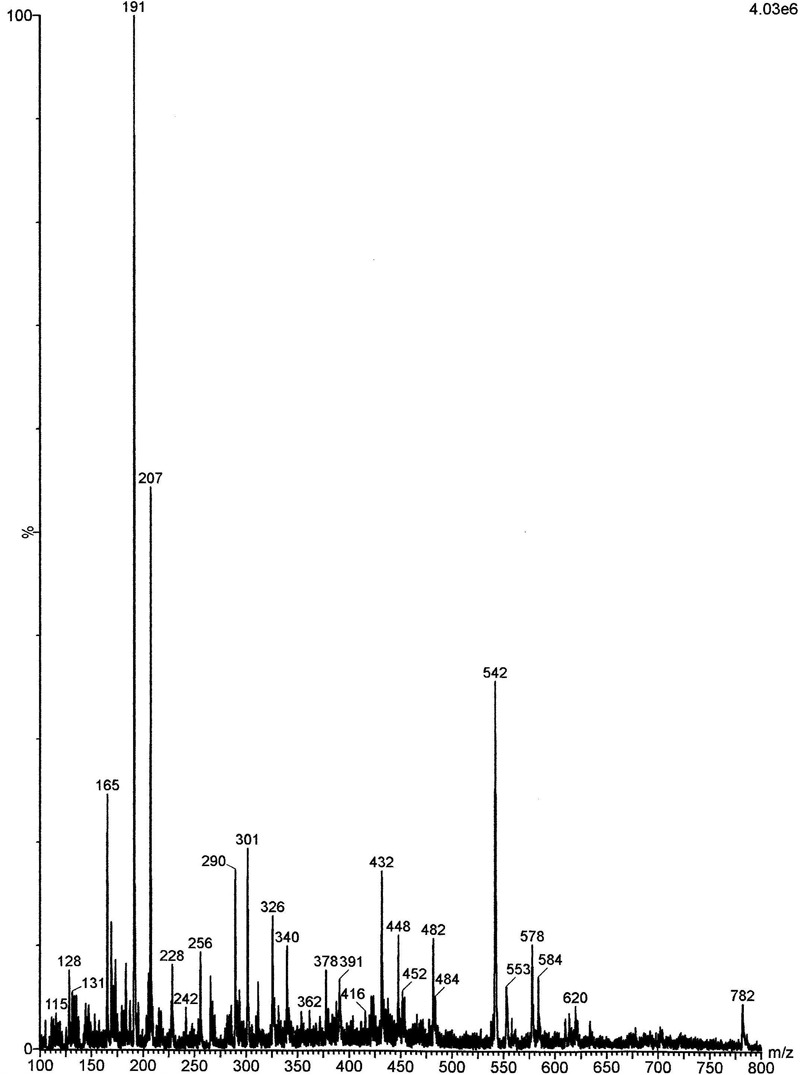
Electrospray ionization mass spectrometry (ESI–MS) of the acetone fraction (AF) obtained from *Buchenavia tomentosa* leaves. (1) Gallic acid (GA) (*m/z* = 169); (2) kaempferol (*m/z* = 285); (3) (-) epicatechin (*m/z* = 289); (4) ellagic acid (*m/z* = 301); (5) vitexin (*m/z* = 431); (6) corilagin (*m/z* = 635).

The analysis by ESI–MS (**Figure 1**) showed the presence of some phenols, such as gallic acid (GA), kaempferol, epicatechin, ellagic acid, vitexin, and corilagin. From the pool of substances found in *B. tomentosa* extracts, GA showed promising anti-candidal activity^12^. For this reason, GA was selected as a molecule of reference in this study.

### Determination of Minimum Inhibitory Concentration and Minimum Fungicidal Concentration of the Acetone Fraction and Gallic Acid

The MICs for all strains of *C. albicans* varied between 0.019 and 2.5 mg ml^-1^ for AF. The MIC_50_ and MIC_90_ values for AF, which are respectively the MIC for the 50 and 90% of the clinical strain samples, were both equal to 0.625 mg ml^-1^. The MIC values for GA varied from 0.625 to 5.0 mg ml^-1^ and MIC_50_ and MIC_90_ were 2.5 and 5 mg ml^-1^, respectively.

For the next tests, we have chosen the highest values of MIC for AF and GA, which were 2.5 and 5.0 mg ml^-1^, respectively.

### Evaluation of the Effects on Virulence Factors of *C. albicans*

#### AF and GA Affect Biofilm Formation by *C. albicans*

**FIGURE 2:**
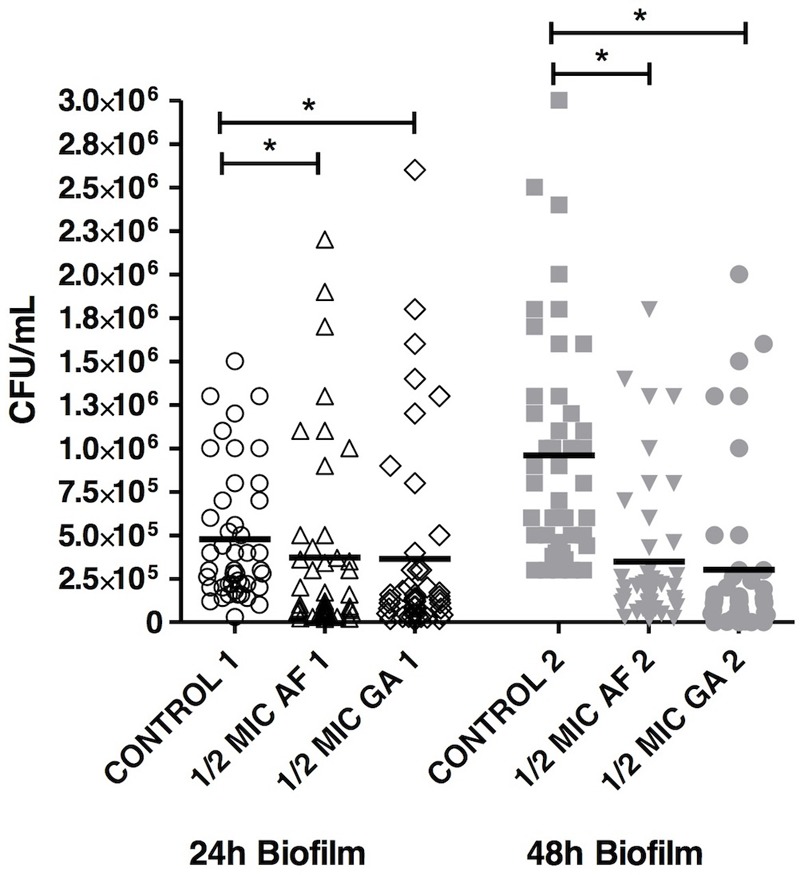
Acetone fraction and GA activities on the formation of *Candida albicans* biofilm; 1/2 MIC: 50% of the inhibitory concentration; Kruskal–Wallis test followed by Dunn’s multiple comparison test. ^∗^*P* < 0.05. Tests were performed in triplicate in three independent experiments using *C. albicans* ATCC 18804, SC5314 and three clinical strains (*n* = 45 for each group).

The results showed that the presence of AF and GA at ½ MIC significantly reduced (*P* < 0.05) the adherence of *C. albicans*. Counts of viable cells in the biofilms were lower in biofilms treated for 24 and 48 h in comparison to the control (non-treated) (**Figure 2**).

#### AF Disrupted 24 and 48-h Preformed Biofilms

**FIGURE 3:**
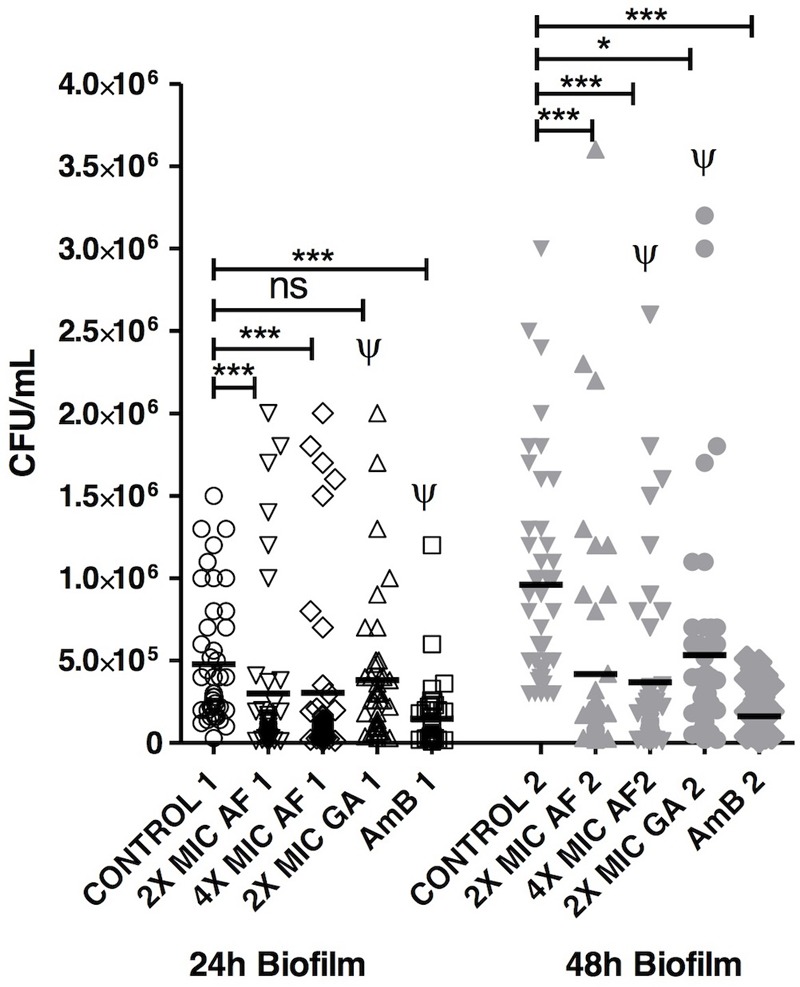
Acetone fraction and GA activities on the eradication of *C. albicans* biofilm; 2 and 4X MIC: 2 and 4 times the minimum inhibitory concentration; AmB, amphotericin B at 2 μg ml^-1^; Kruskal–Wallis test followed by Dunn’s multiple comparison test. ^∗^*P* < 0.05, ^∗∗∗^*P* < 0.0001; ns, not statistically significant. Ψ: Data collected from Teodoro et al. (2017). Tests were performed in triplicate in three independent experiments using *C. albicans* ATCC 18804, SC5314 and three clinical strains (*n* = 45 for each group).

The ability of AF and GA to disrupt preformed biofilms of 24 and 48 h was then investigated and all the treatments reduced the viability of preformed biofilm in comparison to control, apart from GA 2X MIC (24 h) (**Figure 3**). For 24 h-biofilms, there was no statistical difference between 2 and 4X MIC of AF. It is important to notice that there was also no statistical difference between both AF concentrations and AmB, which is one of the reference molecules to treat infections caused by *C. albicans*. This points out to a promising antifungal activity of AF. For comparative purposes, all the data corresponding to GA 2X MIC and AmB (2 μg ml^-1^) were extracted from another study performed by our group (Teodoro et al., 2017). It is important to highlight that all these experiments were performed simultaneously. GA 2X MIC was significantly different from AmB, with *P* < 0.001.

For 48 h-biofilms, which can be considered as mature, AF was efficient in both concentrations and there was also no statistical difference between both (2 and 4X the MIC). These concentrations did not differ from the AmB treatment, reinforcing the potential of AF as an antifungal product. The count of *C. albicans* was also reduced after the treatment with GA 2X MIC in comparison to the control group (*P* < 0.05).

#### AF and GA Showed No Effect on *C. albicans* Enzymatic Activity

**FIGURE 4:**
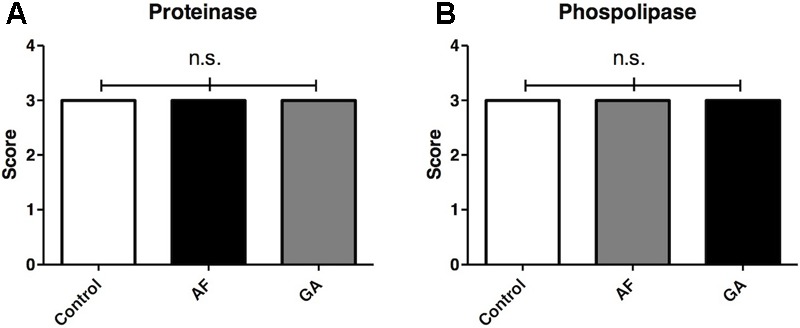
Score representing the activity of control and treatment groups on the production of exoenzymes proteinase **(A)** and phospholipase **(B)** by *C. albicans*. AF and GA were tested at subinhibitory concentration (1/2 MIC). Dunn’s multiple comparison test. Friedman test followed by Dunn’s multiple comparison test; ns, not statistically significant. Tests were performed in triplicate in three independent experiments. Results are expressed as median.

The median value for the enzymatic activity (Pz) of the proteinase was 0.45 for all groups (control, AF and GA), which means a strong positive enzymatic activity (score 3). Statistical analyses did not show a difference between the activity of proteinase for these groups (*P* > 0.05, Friedman/Dunn’s test) (**Figure 4A**). The median values for the enzymatic activity (Pz) of the phospholipase were 0.41, 0.36, and 0.39 (respectively for control, AF and GA), meaning that enzymatic activity was still strong for all groups (score 3). Statistical analyses did not show a difference between the activity of phospolipase for these groups (*P* > 0.05, Friedman/Dunn’s test) (**Figure 4B**).

#### AF and GA Inhibited *C. albicans* Adherence to Oral Epithelial Cells and Morphogenesis

**FIGURE 5:**
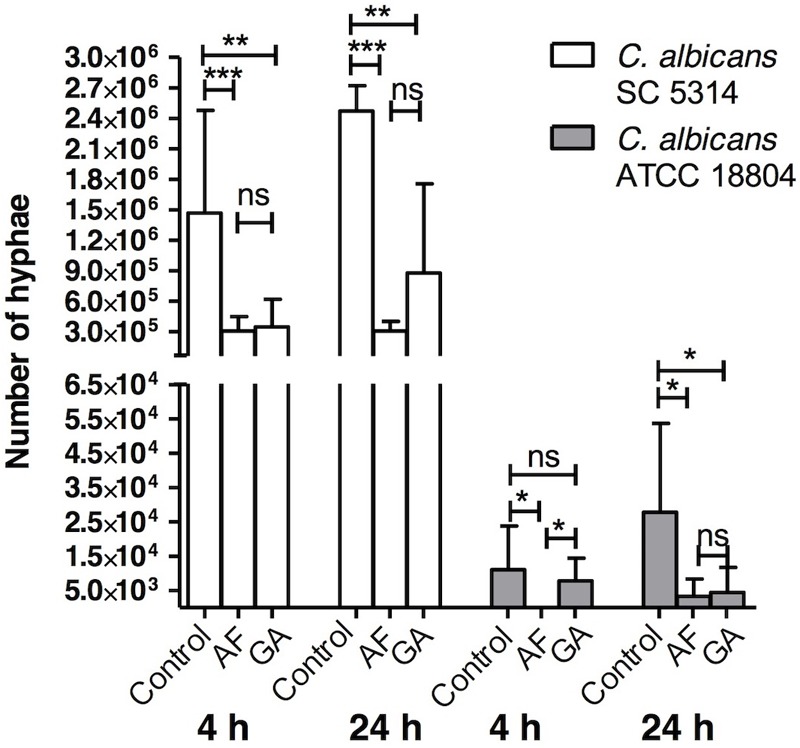
Influence of AF and GA at subinhibitory concentration (half the minimum inhibitory concentration) in hyphae production by *C. albicans* ATCC 18804 and SC 5314; Kruskal–Wallis test followed by Dunn’s multiple comparison test. ^∗^*P* < 0.05; ^∗∗^*P* < 0.001; ^∗∗∗^*P* < 0.0001; ns, not statistically significant. Tests were performed in triplicate in three independent experiments. Results are expressed as mean and SD, *n* = 9 for each box.

Treatments with both AF and GA were effective in reducing the formation of hyphae of *C. albicans* SC 5314 in comparison to the control (**Figure 5**). For the ATCC 18804 strain, the treatment with AF was able to reduce the formation of hyphae after 4 and 24 h. After 4 h, there was a small hyphae formation by ATCC 18804 that was not influenced by the treatment with GA (*P* > 0.05). GA was effective in reducing hyphae from ATCC 18804 only after 24 h (**Figure 5**).

**FIGURE 6:**
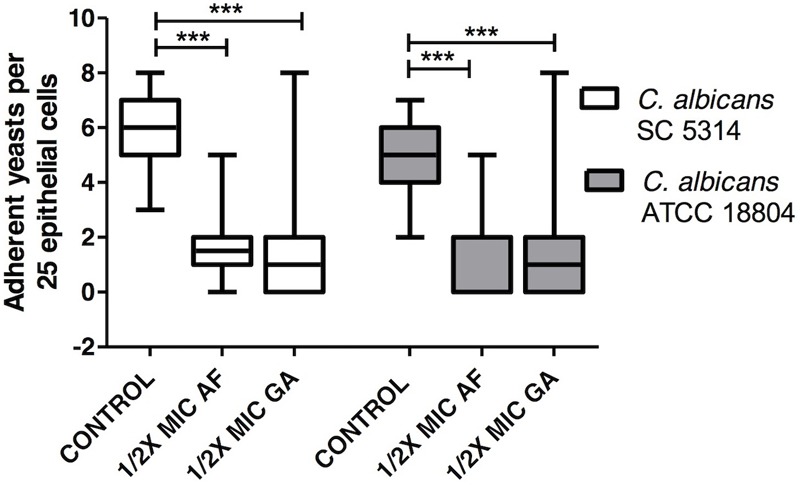
Influence of AF and GA on the adhesion of *C. albicans* ATCC 18804 and SC 5314 to oral epithelial cells; *C. albicans* adhered to 25 epithelial cells were determined by light microscopy at 400X magnification. 1/2 MIC: 50% of the minimum inhibitory concentration. Kruskal–Wallis test followed by Dunn’s multiple comparison test, ^∗∗∗^*P* < 0.0001. Tests were performed in decuplicate in five independent experiments. The results are expressed as mean and SD, *n* = 50 for each box.

Treatments with AF and GA decreased adherence of *C. albicans* strains ATCC 18804 and SC 5314 (*P* < 0.001) to oral epithelial cells (**Figure 6**).

#### AF and GA Showed Slight to Moderate Cytoxicity

**FIGURE 7:**
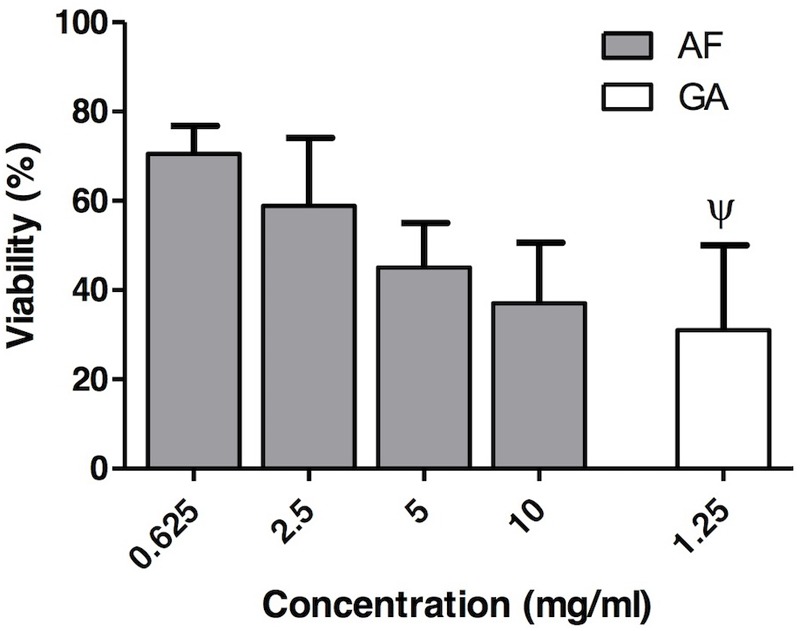
*In vitro* cytotoxicity assay of the AF (gray boxes) at different concentrations (0.625, 2.5, 5, and 10 mg ml^-1^) and GA (white box) (1.25 mg ml^-1^). Results are expressed as mean and SD, *n* = 6 for each box. Ψ: Data collected from Brighenti et al. (2017).

The values of cell viability after AF treatment at MIC_90_ (0.625 mg ml^-1^), at 2.5 mg ml^-1^ (the highest value of MIC for AF), at 5 mg ml^-1^ (2X MIC) and at 10 mg ml^-1^ (4X MIC) were, respectively, 70.5 ± 6.3, 58.9 ± 15.2, 45.0 ± 10.9, and 37.0 ± 13.6% (**Figure 7**). For GA the cell viability at 1.25 mg ml^-1^ was 31 ± 19%. This result was obtained from Brighenti et al. (2017).

## Discussion

The AF from *B. tomentosa* aqueous extract presented antifungal activity on *C. albicans*. This fraction inhibited some *C. albicans* virulence factors, such as the ability to form biofilm, to change its morphology (from yeasts to hyphae), and to adhere to epithelial cells.

The analysis of AF by ESI–MS (**Figure 1**) showed a similar profile to the one obtained in previous works, which used aqueous (Teodoro et al., 2015a) and ethanolic extracts (Brighenti et al., 2017) from *B. tomentosa*. All these extracts showed the presence of some phenols, which are chemical compounds related to a potential antimicrobial activity (Teodoro et al., 2015b). In this present study, we performed the extraction with acetone, since this solvent can concentrate most efficiently the phenol content and thus increase the antimicrobial effect (Alberti et al., 2014).

It is interesting to notice that in previous studies the MICs for *C. albicans* ATCC 18804 of aqueous and ethanol 99° extracts were, respectively, 12.5 (Teodoro et al., 2015a) and 0.4 mg ml^-1^ (Brighenti et al., 2017). In this present study, the MIC for *C. albicans* ATCC 18804 was much lower (0.019 mg ml^-1^), most probably due to the higher concentration of phenols present in AF compared to aqueous and ethanolic extracts. The extraction of phenols depends on the polarity of the solvent, as well as on the corresponding solubility of the compounds (Kchaou et al., 2013). In this present study, acetone seemed to have a more adequate polarity and solubility to extract higher amounts of phenol compounds than the other previously used solvents, such as water (Teodoro et al., 2015a) or ethanol 99° (Brighenti et al., 2017).

Gallic acid was the phenol chosen in this study to investigate the antifungal activity as a molecule of reference of the AF fraction. It was selected due to its promising anti-candidal activity (Alves et al., 2014; Teodoro et al., 2015a). The mechanism of action of GA is not fully understood. Li et al. (2017) recently reported that GA reduced the activity of sterol 14α-demethylase P450 (CYP51), that is a cytochrome P450 enzyme involved in the biosynthesis of sterols (Hargrove et al., 2017). The values of MICs for GA are higher than the values for AF, suggesting that GA is not the sole responsible for the antifungal activity of AF, which is mainly due to a synergism between the compounds present in this extract.

Acetone fraction and GA were able to inhibit the formation and to disrupt *C. albicans* biofilms (**Figure 2**). For GA, only one concentration (2X the MIC) was used, since 4X the MIC (i.e., 20 mg ml^-1^) is insoluble in water (Srinivas et al., 2010). A study performed by our group (Teodoro et al., 2017) improved the GA solubility using cyclodextrins, which increase the aqueous solubility. These molecules present a lipophilic cavity that hosts the water insoluble drug, and a hydrophilic exterior, which increases the aqueous solubility.

Surprisingly, GA at 2X MIC was able to reduce the 48 h-biofilm (**Figure 2**), without effect on the 24 h-biofilm. The authors previously hypothesized (Teodoro et al., 2017) that this phenomenon could be due to the dynamics of biofilm formation. In the biofilm of 24 h, the composition of cells, molecular structures, and water are different from the biofilm of 48 h, which could interfere on the efficiency of GA. Additionally, still according to Teodoro et al. (2017), GA might act on matrix compounds of biofilms, which are quantitatively more present in the 48 h biofilms.

Neither AF nor GA was able to reduce the proteinase and phospholipase productions by *C. albicans*, suggesting that these compounds are not able to act on these virulence factors of *C. albicans*. These results are in accordance with Li et al. (2001), which have shown that GA did not have an effect on the production of proteinases by *C. albicans*.

Acetone fraction and GA inhibited the formation of hyphae (**Figure 5**). Two reference strains of *C. albicans* were used in order to investigate the interspecies variability. As shown in **Figure 5**, and also in the literature (Hirakawa et al., 2015), SC5314 forms more hyphae than other strains. The inhibition of hyphae formation is an encouraging result, in particular considering the effect on a hyper filamentous strain (SC 5314). Filamentation is considered a crucial virulence factor in *C. albicans* (Lu et al., 2014), as the hyphae form is related to increased tissue invasion of the host. Also, an attenuation of the *in vivo* virulence was observed as consequence of blocking filamentation (Saville et al., 2006). For this reason, substances or therapies targeting morphogenesis have been considered promising therapeutic options (Jacobsen et al., 2012).

Both treatments, AF and GA, decreased the adherence of *C. albicans* to oral epithelial cells. This finding is interesting since the adhesion on mucosal surfaces is a first step to start an infectious disease (Gow et al., 2012). There are some adhesins on the cell surface of *C. albicans* that bind to host surfaces. Hyphal wall protein 1 (Hwp1, encoded by the HWP1 gene) and the agglutinin-like sequence (ALS) family are widely studied adhesins and have been shown as important for the colonization by *C. albicans* in oral epithelial cells (Sundstrom et al., 2002). Further studies should be performed to investigate the AF and GA activity on these adhesins.

Acetone fraction (70.5 ± 6.3%) and GA (31 ± 19%) were, respectively, slightly and moderate cytotoxic according to Sletten and Dahl (1999) classification. For AF, even at the highest tested concentration (10 mg ml^-1^), the cytotoxicity was moderate (cell viability equal to 37.0 ± 13.6%), according to the aforementioned classification. For GA the cell viability was tested at 1.25 mg ml^-1^, which is lower than MIC_90_ (5 mg ml^-1^). It was not possible to perform the cytotoxicity test with higher concentrations of GA, since above this concentration (1.25 mg ml^-1^) the GA reacts with the medium (DMEM) forming a dark coloration that biases the interpretation of results, since the methodology is colorimetric. Sletten and Dahl (1999) classified as severely toxic a compound presenting a cell viability lower than 30%. Considering that the standard deviation is close to 20%, GA is in the limit between moderately and severely toxic. It is important to emphasize that, in the present study, only one cell line was used (Vero cells). Future studies with other cell lines, such as keratinocytes, should be performed in order to validate the use of AF for treatment of superficial candidosis. These results reinforce that the AF, which contain a combination of several molecules, presents a greater potential of antimicrobial activity than its isolated molecule, the GA.

## Conclusion

The AF presented the best antifungal activity among several extracts from *B. tomentosa*. Both AF and its isolated compound, the GA, presented activity on *C. abicans*; nonetheless, AF presented lower values of MIC and was less cytotoxic than GA. This study suggested that both, AF and GA, act on *C. albicans* reducing its virulence factors, such as ability to form biofilms, hyphae and to adhere on oral epithelial cells. Further studies using animal models are needed. The present work can contribute to the development of new products for the treatment of candidiasis.

## Author Contributions

GT, AG, MS, AD, ÁdD, FB, and CK-I designed the research. GT, AG, AD, ÁdD, MT, and FB performed the experiments. AG, MS MT, and CK-I analyzed the data. GT, AG, and CK-I wrote the paper. GT, AG, MS, MT, AD, ÁdD, FB, and CK-I critically revised the paper.

## Conflict of Interest Statement

The authors declare that the research was conducted in the absence of any commercial or financial relationships that could be construed as a potential conflict of interest.

## Supplementary Material

FIGURE S1 Fractions obtained from aqueous extract from *Buchenavia tomentosa*.
