# Supplementary figures and images for: Effects of Acetone Fraction From Buchenavia tomentosa Aqueous Extract and Gallic Acid on Candida albicans Biofilms and Virulence Factors
Source: Front Microbiol. 2018 Apr 5;9:647. doi: 10.3389/fmicb.2018.00647 (PMC5895766; doi:10.3389/fmicb.2018.00647)

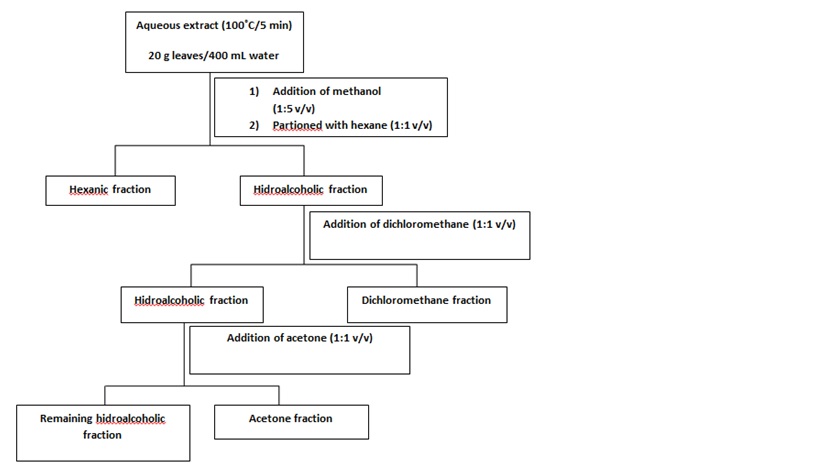

Supplement: FIGURE S1 — Fractions obtained from aqueous extract from Buchenavia tomentosa. [file Image_1.JPEG]
